# Supplementary material for: HIVprotI: an integrated web based platform for prediction and design of HIV proteins inhibitors
Source: J Cheminform. 2018 Mar 9;10:12. doi: 10.1186/s13321-018-0266-y (PMC5845081; doi:10.1186/s13321-018-0266-y)
Supplement: Supplementary file 2 — Additional file 2. Source code of HIVProtI web server. [file 13321_2018_266_MOESM2_ESM.zip › HIVprotI_Source-code/hivprotI/weka-3-6-6/doc/weka/gui/graphvisualizer/DotParser.html]

DotParser


---


|  |  |  |
| --- | --- | --- |
| **Overview** \| **Package** \| **Class** \| **Tree** \| **Deprecated** \| **Index** \| **Help** \| **Weka's home** | |  |
| **PREV CLASS**   **NEXT CLASS** | **FRAMES**    **NO FRAMES**     **All Classes** |  |
| SUMMARY: NESTED \| FIELD \| CONSTR \| METHOD | DETAIL: FIELD \| CONSTR \| METHOD |  |


---


## weka.gui.graphvisualizer Class DotParser

```
java.lang.Object
  weka.gui.graphvisualizer.DotParser
```

**All Implemented Interfaces:**: GraphConstants

---

``` public class DotParser extends java.lang.Object implements GraphConstants ```

This class parses input in DOT format, and
builds the datastructures that are passed to it.
It is NOT 100% compatible with the DOT format. The
GraphNode and GraphEdge classes do not have any provision
for dealing with different colours or shapes of nodes,
there can however, be a different label and ID for a
node. It also does not do anything for labels for
edges. However, this class won't crash or throw an
exception if it encounters any of the above
attributes of an edge or a node. This class however,
won't be able to deal with things like subgraphs and
grouping of nodes.

**Version:**
:   $Revision: 7059 $ - 23 Apr 2003 - Initial version (Ashraf M. Kibriya)

**Author:**
:   Ashraf M. Kibriya (amk14@cs.waikato.ac.nz)

---

**Field Summary**

**Fields inherited from interface weka.gui.graphvisualizer.GraphConstants**

`DIRECTED, DOUBLE, NORMAL, PLURAL_DUMMY, REVERSED, SINGULAR_DUMMY`


**Constructor Summary**

`DotParser(java.io.Reader input,
FastVector nodes,
FastVector edges)`
  
          Dot parser Constructor


| **Method Summary** | |
| --- | --- |
| `java.lang.String` | `parse()`             This method parses the string or the InputStream that we passed in through the constructor and builds up the m\_nodes and m\_edges vectors |
| `static void` | `writeDOT(java.lang.String filename, java.lang.String graphName, FastVector nodes, FastVector edges)`             This method saves a graph in a file in DOT format. |

**Methods inherited from class java.lang.Object**

`equals, getClass, hashCode, notify, notifyAll, toString, wait, wait, wait`

**Constructor Detail**

### DotParser

```
public DotParser(java.io.Reader input,
                 FastVector nodes,
                 FastVector edges)
```

:   Dot parser Constructor

    **Parameters:**: `input` - - The input, if passing in a string then encapsulate that in String reader object: `nodes` - - Vector to put in GraphNode objects, corresponding to the nodes parsed in from the input: `edges` - - Vector to put in GraphEdge objects, corresponding to the edges parsed in from the input


**Method Detail**

### parse

```
public java.lang.String parse()
```

:   This method parses the string or the InputStream that we
    passed in through the constructor and builds up the
    m\_nodes and m\_edges vectors

    :   **Returns:**: - returns the name of the graph

---


### writeDOT

```
public static void writeDOT(java.lang.String filename,
                            java.lang.String graphName,
                            FastVector nodes,
                            FastVector edges)
```

:   This method saves a graph in a file in DOT format.
    However, if reloaded in GraphVisualizer we would need
    to layout the graph again.

    :   **Parameters:**: `filename` - - The name of the file to write in. (will overwrite): `graphName` - - The name of the graph: `nodes` - - Vector containing all the nodes: `edges` - - Vector containing all the edges


---


|  |  |  |
| --- | --- | --- |
| **Overview** \| **Package** \| **Class** \| **Tree** \| **Deprecated** \| **Index** \| **Help** \| **Weka's home** | |  |
| **PREV CLASS**   **NEXT CLASS** | **FRAMES**    **NO FRAMES**     **All Classes** |  |
| SUMMARY: NESTED \| FIELD \| CONSTR \| METHOD | DETAIL: FIELD \| CONSTR \| METHOD |  |


---
